# Urinary Incontinence in Hospitalised Elderly Patients: Do Nurses Recognise and Manage the Problem?

**DOI:** 10.1155/2011/671302

**Published:** 2011-04-06

**Authors:** Sabin Zürcher, Susi Saxer, René Schwendimann

**Affiliations:** ^1^Lindenhofspital, Bremgartenstrasse 117, 3012 Bern, Switzerland; ^2^Institute of Applied Nursing Science, University of Applied Sciences of St. Gallen, Rosenbergstrasse 22, 9001 St. Gallen, Switzerland; ^3^Institute of Nursing Science, University of Basel, Bernoullistrasse 28, 4956 Basel, Switzerland

## Abstract

This study examined to what extent nurses recognize urinary incontinence (UI) in elderly hospital patients, what UI interventions nurses realize, and if elderly inpatients are willing to raise the topic during their hospital stay. A convenience sample of 78 elderly inpatients in a Swiss hospital were screened for UI and asked if they were willing to be questioned about UI during hospitalisation. Nursing records were analysed as to whether UI had been recognized, and to collect data on interventions. Forty-one patients (51%) screened positive for UI, of whom 10 (24%) were identified as such in their nursing records. The single intervention documented was the use of incontinence pads. Only 5 patients preferred not to be asked about UI at hospital. Nurses in the study hospital should systematically ask elderly patients about UI and provide them with information on interventions.

## 1. Introduction

Urinary incontinence (UI) is defined by the International Continence Society [1] as “any involuntary leakage of urine”. Depending on study populations and definitions used, its prevalence varies between 8% and 72%, among people aged 65 and older [2]. In acute hospital settings, 35% to 42% of adult patients are affected by UI [3, 4]. UI can negatively impact patients' physical and mental health and quality of life [5]. Roughly half of the persons suffering from UI never seek professional help [6], often due to inappropriate beliefs such as “UI is a normal part of ageing” or “UI cannot be treated” [6, 7]. In fact, with appropriate management, most cases of UI in older patients can be improved or cured [8]. While a hospital stay represents an opportunity to identify and appropriately address UI, only 10% to 59% of patients with UI are identified during hospitalization [3, 9]. Even then, nursing records contain little UI assessment information [9, 10]. Interventions are documented in only approximately half of identified cases and mainly reflect passive measures, such as the use of incontinence pads [9–11]. 

Since elderly people constitute a growing hospital population, evaluation and improvement of this patient group's quality of care is recognized as a priority in the study hospital. Among elements of geriatric care, UI was chosen as the subject of this investigation, because of its high prevalence, its impact on quality of life, and because hospitalisation represents a good chance to discover it and offer patients interventions [9]. The purpose of this study was to evaluate nursing practices regarding elderly hospitalised inpatients with UI in order to determine the need for quality improvement or redirection of care. 

This study focused on the following research questions: (1) To what extent nurses recognize UI in elderly inpatients?, (2) What interventions were carried out by the nurses to manage UI?, and (3) Is UI a topic elderly people are willing to be questioned about at hospital?

## 2. Design, Setting, and Sample

In this cross-sectional study, a convenience patient sample was used. During a six weeks time period in spring 2007, it included all consecutively admitted patients to the units of Internal Medicine and Orthopaedics of a private, nonprofit 250-bed urban general hospital in Switzerland. In this hospital, patients aged 65 and older constitute approximately one third of the patient population. However, screening for incontinence is not routinely performed, no specific standard exists for incontinence care, and no specialised incontinence nurse is available. Patient inclusion criteria for the study were: age 65 and older, hospitalisation for at least 48 hours, the ability to understand, speak, and read German, and a health status allowing participation in a conversation. Patients with renal failure or medically diagnosed UI were excluded.

## 3. Variables and Measurement

### 3.1. Screening for UI

To determine the number of patients with UI, a screening procedure was performed using the following questions, developed by the Association of Women's Health, Obstetric and Neonatal Nursing [12]:

- Do you ever leak urine when you do not want to?
- Do you ever leak urine when you cough, laugh, or exercise?
- Do you ever leak urine on the way to the bathroom?
- Do you ever use pads, tissue, or cloth in your underwear to catch urine?

Responses were either yes or no. Patients with at least one positive answer were considered to be incontinent of urine. This screening instrument is recommended in the expert standard on continence designed by the German Network of Quality Development in Nursing (Deutsches Netzwerk für Qualitätsentwicklung in der Pflege, [13]).

### 3.2. Nursing Records

Nursing records served as the data source for gender, age, and length of stay. The records of positively screened patients were checked for any indications of nurses′ identification of UI and management interventions. UI was considered to have been identified by the nurses if the nursing record included (a) documentation of UI, (b) a note to involve a physician for diagnosis or treatment of UI, or (c) a documented plan for a nursing intervention.

### 3.3. Patients' Expectations

All participating patients were asked whether they were willing to be interviewed about UI at the hospital (yes/no/indifferent), and, if yes, by whom (nurse/physician/indifferent). Positively screened patients were additionally asked whether they wished to improve their situation regarding UI (yes/no).

## 4. Data Collection

Patient recruitment occurred over the course of a six weeks study period in spring 2006 by the first author, who screened for UI face to face and asked patients about their expectations. Participants' nursing records were then reviewed using a checklist covering the following topics: nursing history and assessment, care plan, discharge plan, and interdisciplinary communication/prescriptions.

## 5. Ethical Considerations

All participants were informed about the purpose and methods of the study. Participation was voluntary, and confidentiality was guaranteed. Written consent was obtained from all participants. Patients with UI were offered information on their condition, on treatment options, and on respective contact addresses in their areas. 

The study was approved by the Ethics Review Board of the Canton of Berne.

## 6. Data Analysis

Continuous data were described using mean and standard deviation. Frequencies were calculated for nominal data. We also tested (1) for associations between the presence of nursing record entries on UI and patients' wishes to improve their condition, (2) whether continent and incontinent patients differed regarding their willingness to be questioned about UI, and (3) by whom they would prefer to be asked about their condition. We initially used logistic regression to test these three questions, thereby controlling for gender and unit. However, because gender and unit were no significant confounders, we alternatively performed reported Fisher's exact tests and reported the results of this test. Data analyses were performed in SPSS version 11.0 (SPSS Inc., Chicago, IL, USA).

## 7. Results

### 7.1. Sample Characteristics

During the recruitment period, a total of 189 patients 65 years and older were admitted to the two designated study wards. Of the 81 who fulfilled the inclusion criteria, 78 agreed to participate and 3 refused. No patient was excluded because of a medically diagnosed UI. Thirty-one (40%) participants were from Internal Medicine and 47 (60%) from Orthopaedics. Female participants (*n* = 53; 68%) showed a slightly higher UI prevalence than men (57% versus 43%; *P* = .34). The mean age was 75 years (SD 7.7) and the mean length of hospital stay was 13 days (SD 9.8). UI screening showed an overall prevalence rate of 53%  (*n* = 41), with incontinent patients slightly older than continent patients (76 versus 74 years).  Table 1 shows the characteristics of incontinent and continent patients in detail. 

### 7.2. Recognition of UI by Nurses

Of the 41 patients who screened positive for UI, the nursing records identified 10 (24%), via either direct references to UI in the nursing histories (*n* = 4), documented interventions (*n* = 4), or both (*n* = 2). UI indicators are summarized in Table 2. 

### 7.3. Nursing History and Management Interventions for UI

None of the 6 nursing histories that identified UI contained conclusive UI assessments, as no more than two assessment factors were documented for any patient. Factors documented were severity of UI symptoms (*n* = 2), duration of UI (*n* = 1), onset of UI (*n* = 1), type of UI (*n* = 2), and patients' UI self-management strategies (*n* = 2). No assessment reported the patient's degree of discomfort or desire for change. 

Interventions were documented for 6 patients, all relating to the use of absorbent products. No records could be found on long-term interventions such as counselling or referrals to special care. Only 2 of the 6 patients identified in the nursing history as incontinent received recorded UI interventions.

### 7.4. Patients' Expectations

Of the 41 UI-affected patients, 18 (44%) expressed wishes to improve their continence (Table 3). We found no association between patients' wishes to improve their UI situation and the presence of related entries in the nursing records (*P* = .17). Thirty-five of the 41 incontinent patients (85%) and 25 of the 37 continent patients (68%) said they were willing to be questioned about UI at the hospital. No significant difference was found among continent and incontinent patients (*P* = .15). Whether they were approached by a nurse or a physician regarding UI did not matter to more than half of patients (*P* = .50). Among patients expressing a preference, nurses were given priority over physicians (72%); however, this difference was not significant (*P* = .24). 

## 8. Discussion

In this study, UI was shown to affect half of elderly hospitalised patients. Despite the fact that a considerable proportion of the sample screened positive for UI, only a quarter of this group had entries in their nursing records identifying it. Even less had interventions documented for UI, and all of these were related to short-term inpatient management. These findings are in line with previous studies, showing that nurses in acute care settings recognize and manage UI poorly in their patients [3, 9, 14]. 

The UI prevalence rate of 51% found in this study is consistent with the literature [2–4]. Though gender differences were not significant, the trend of higher UI rates in women is also in accordance with other reports [2, 3]. At 24%, the rate of nurse identification of UI was low, compared to 45% reported by Wagg et al. [10]. Systematic screening could enhance identification of UI and can be expected to be well accepted by patients, as, in our study, most participants—whether continent or incontinent—said they were willing to be questioned about UI at the hospital. According to our findings, nurses are well placed to approach the issue: 82% of participants stated either that they had no preference as to who approached them regarding UI or that they would prefer a nurse. 

Analysis of patients' records indicates that UI is not an integral part of nursing histories in the study hospital. Even in cases where UI was recognized, it seemed haphazard if a further assessment was made and what factors were included. A similar lack of systematic assessment of UI has been observed in other studies [9, 10, 14]. 

The number of interventions initiated in our sample is much lower than in similar studies, where half of the patients received care plans for UI [9, 10]. For the current study, the only nursing intervention shown in the patient documentation was the use of absorbent products. Other authors have reported finding records of various management methods, such as fixed-interval toileting, but these have frequently proven inappropriate [9, 10, 14]. Wagg et al. [10] and Palese et al. [11] both cite containment as the most frequent nursing intervention. Unlike interventions to manage UI actively, absorbent product use is not inadequate per se [11], but it is insufficient for patients who desire to improve their situation. 

Regarding the nurses' poor recognition and management of UI, possible causes are discussed in the literature: nurses may not perceive UI management as part of their acute care duty; they may have knowledge deficits and feel uncertain about UI assessment and management; and they may have misconceptions about normal ageing and treatment prospects [14]. Other causes mentioned are time constraints, nurse-patient ratios, and care delegated to unlicensed assistive personnel [11]. 

### 8.1. Limitations

This study's rather small convenience sample from a single hospital prevents generalization of its findings to a broader elderly inpatient population, even though its results are partially confirmed by similar research. A further limitation of our study is that a lack of documented observations and interventions regarding UI does not automatically imply the lack of UI management, as nurses may not have reported all their observations or actions. Identification rate of UI and the number of interventions might be higher in practice than the records reflect. As a contrary argument, staff awareness of UI may actually have increased during the study period, with nurses making more record entries on the topic than usual. However, the fact that the lack of any systematic approach to assessing UI was also observed in other studies adds to the credibility of our findings [9–11, 14].

### 8.2. Implications for Nursing Practice

In order for nurses to systematically identify UI and initiate long-term management, various measures can be taken. First, nursing education in UI could improve associated care. From research, it is known that what was learned (knowledge) and how the individual felt about something (attitude) influence how someone acts (practice) [15, 16]. Also, in order to enhance UI recognition, screening should be integrated into the standard nursing history with an item on urination added to preprinted nursing history charts. The screening instrument used should contain a trigger question to elicit whether patients affected wish to improve their UI situation. Nurses should actively identify and counsel such patients. The goals of counselling must be for the patient to realise that UI is not an inevitable part of ageing and to know where to seek treatment. Franzén et al. showed that providing information about UI and its management options is an effective method to motivate affected persons to initiate treatment [17]. Considering the usually short hospitalisation period and the priority of other health problems, the authors suggest that in an acute care setting it is more realistic for nurses to focus their long-term management interventions on counselling rather than on initiating treatment. 

Implementation of a quality improvement programme for UI requires consideration of the strength and weaknesses in the organization, such as personnel resources, staff expertise, time, and reimbursement pressures.

## 9. Conclusions

The relatively poor recognition of UI in elderly patients by the acute care nurses in this study highlights the need for the integration of an incontinence screening in standard nursing assessment. Such a strategy is supported since the majority of elderly incontinent patients would like to raise the topic of UI at hospital and could be offered advice for UI management by the nurse. Quality of nurses' incontinence management needs to be improved and should focus on patient counselling for long-term management.

## Figures and Tables

**Table 1:** Patients' characteristics.

	Incontinent (*n* = 41)		Continent (*n* = 37)	
Gender				
Women	30	(56.6%)	23	(43.4%)
Men	11	(44.0%)	14	(56.0%)
Mean age in years	76	(7.7 SD)	74	(7.6 SD)
Mean length of stay in days	12.5	(8.5 SD)	13.4	(11.1 SD)
Units				
Internal Medicine	18	(58.1%)	13	(41.9%)
Orthopaedics	23	(48.9%)	24	(51.1%)

**Table 2:** Signs of identification of UI in the nursing records of incontinent patients (*n* = 41).

No entries	31	(75.6%)
Entry about UI	10	(24.4%)
Prescription/communication sheet	0	
Nursing history/assessment	6	(14.6%)
Care plan	0	
Single intervention planned	6	(14.6%)
Discharge plan	0	

**Table 3:** Patients' expectations.

	Incontinent (*n* = 41)		Continent (*n* = 37)	
Do you wish to improve your situation referring to UI?				
Yes	18	(43.9%)		
No	23	(56.1%)		
Would you like to be asked about UI at the hospital?				
Yes	35	(85.4%)	25	(67.6%)
No	1	(2.4%)	4	(10.8%)
Indifferent	5	(12.2%)	8	(21.6%)
By whom would you like to be asked?				
Nurse	13	(31.7%)	8	(21.6%)
Physician	3	(7.3%)	5	(13.5%)
Indifferent	24	(58.5%)	20	(54.0%)
No answer	1	(2.4%)	4	(10.8%)

## References

[1] International Continence Society The standardisation of terminology in lower urinary tract function: report from the standardisation sub-committee of the International Continence Society. http://www.icsoffice.org/Documents/Documents.aspx?DocumentID=605.

[2] Hunskaar S, Burgio K, Clark A, Abrams P, Cardozo L, Khoury S, Wein A(Hrsg) (2005). Epidemiology of urinary and faecal incontinence and pelvic organ prolapse. *Proceedings of the 3rd International Consultation on Incontinence, Paris, June 2004*.

[3] Schultz A, Dickey G, Skoner M (1997). Self-report of incontinence in acute care. *Urologic Nursing*.

[4] da Silva AP, Santos VL (2005). Prevalence of urinary incontinence in hospitalized patients. *Revista da Escola de Enfermagem da U S P.*.

[5] Ko Y, Lin SJ, Salmon JW, Bron MS (2005). The impact of urinary incontinence on quality of life of the elderly. *American Journal of Managed Care*.

[6] Teunissen D, Lagro-Janssen T (2004). Urinary incontinence in community dwelling elderly: are there sex differences in help-seeking behaviour?. *Scandinavian Journal of Primary Health Care*.

[7] Dugan E, Roberts CP, Cohen SJ (2001). Why older community-dwelling adults do not discuss urinary incontinence with their primary care physicians. *Journal of the American Geriatrics Society*.

[8] Agency for Health Care Policy and Research Urinary incontinence in adults: acute and chronic management. http://www.ncbi.nlm.nih.gov/.

[9] Cheater FM (1993). Retrospective document survey: identification, assessment and management of urinary incontinence in medical and care of the elderly wards. *Journal of Advanced Nursing*.

[10] Wagg A, Lowe D, Peel P, Potter J (2008). Continence care for older people in England and Wales: data from a national audit. *Journal of Wound, Ostomy and Continence Nursing*.

[11] Palese A, Regattin L, Venuti F (2007). Incontinence pad use in patients admitted to medical wards: an Italian multicenter prospective cohort study. *Journal of Wound, Ostomy and Continence Nursing*.

[12] Association of Women’s Health Obstetric and Neo-natal Nurse Continence for women. Evidence-based practice guideline. http://www.guideline.gov/.

[13] Deutsches Netzwerk fur Qualitatsentwicklung in der Pflege (2006). Expertenstandard Forderung der Harnkontinenz in der Pflege einschliesslich Kommentierung und Literaturanalyse (Sonderdr. ed.). Osnabruck: Fachhochschule Osnabruck.

[14] Cooper G, Watt E (2003). An exploration of acute care nurses’ approach to assessment and management of people with urinary incontinence. *Journal of Wound, Ostomy and Continence Nursing*.

[15] Henderson SJ, Schmidt Kashka M, Larson A (2000). Effect of knowledge, attitude and belief on nurses’ practice regarding urinary incontinence in adults. *Urological Nursing*.

[16] Vinsnes A, Harkless G, Haltbakk G, Bohm J, Hunskaar S (2001). Healthcare personnel’s attitudes towards patients with urinary incontinence. *Journal of Clinical Nursing*.

[17] Franzén K, Johansson JE, Andersson G, Nilsson K (2008). Urinary incontinence: evaluation of an information campaign directed towards the general public. *Scandinavian Journal of Urology and Nephrology*.

